# Assessing the Applicability of Sentinel-1 D-InSAR for Snow Depth Retrieval in the Maxian Mountains

**DOI:** 10.3390/s26206525

**Published:** 2026-10-15

**Authors:** Tianwen Feng, Jingwei Zhu, Zhenhua Cai, Wenxin He

**Affiliations:** 1 Faculty of Geomatics, Lanzhou Jiaotong University, Lanzhou 730070, China; 2 National-Local Joint Engineering Research Center of Technologies and Applications for National Geographic State Monitoring, Lanzhou 730070, China; 3 School of Geography, Qinghai Normal University, Xining 810008, China; hewenxin@nieer.ac.cn

**Keywords:** Sentinel-1, snow depth, D-InSAR, uncertainty analysis

## Abstract

**Highlights:**

**What are the main findings?**

- Validation using UAV-LiDAR data showed mean absolute error (MAE) values of 1.25 cm and 3.80 cm for the November and April retrievals, respectively.
- Low coherence increased retrieval uncertainty, and snow density had a stronger effect than local incidence angle.

**What are the implications of the main findings?**

- D-InSAR is more reliable for thin, dry snow under high-coherence conditions.
- Coherence screening and snow density constraints can improve retrieval reliability.

**Abstract:**

Differential interferometric synthetic aperture radar (D-InSAR) provides a potential approach for retrieving mountain snow depth at high spatial resolution using Sentinel-1 interferometric phase information. However, its performance in complex mountainous terrain remains insufficiently evaluated. This study assessed the applicability and uncertainty of Sentinel-1 D-InSAR for multi-temporal snow depth retrieval in the Maxian Mountains. Sentinel-1 interferometric pairs from 2020 to 2022 were used for snow depth retrieval, and UAV-LiDAR snow depth estimates served as independent reference data. The spatial distribution of interferometric coherence was examined, and coherence statistics were compared among elevation intervals and land-cover types. Snow depth was derived from interferometric phase using snow density and local incidence angle as model inputs, and sensitivity to both parameters was evaluated. The retrievals characterized the main spatial distribution of snow depth, but validation accuracy varied markedly among periods. Compared with November 2020, the root mean square error (RMSE) and mean absolute error (MAE) in April 2021 increased by 3.62 and 2.55 cm, respectively, indicating larger errors under spring snow conditions. Interferometric coherence was a key factor controlling retrieval reliability, and areas with low coherence were more prone to phase-unwrapping errors, missing retrievals, and local anomalies. Sensitivity analysis showed that snow density had a greater influence than local incidence angle.

## 1. Introduction

Mountain snow is an important component of seasonal water resources [1,2]. Snow depth is a key variable for characterizing snow accumulation [3]. In mountainous areas, the spatial distribution of snow depth is highly heterogeneous because of differences in elevation, slope, aspect, wind redistribution, and local terrain conditions [4]. Traditional snow depth measurements can provide accurate in situ data, but the number of observation sites is usually limited, making it difficult to fully characterize spatial variations in mountain snow depth [5]. Optical remote sensing is useful for monitoring snow cover extent, but it is often affected by cloud cover and illumination conditions [6]. Passive microwave remote sensing has strong capability for regional snow monitoring, but its coarse spatial resolution limits its application in complex mountainous terrain [7].

Synthetic aperture radar (SAR) can acquire surface information under cloud cover and provides relatively high spatial resolution [8]. In recent years, continuous C-band observations from Sentinel-1 have advanced research on SAR snow depth retrieval [9]. Previous studies have shown that C-band backscatter, especially cross-polarized backscatter and polarization combinations, is sensitive to changes in mountain snow accumulation and has been used for snow depth mapping in mountainous regions of the Northern Hemisphere and the European Alps [10,11]. Subsequent studies have extended this method to sub-kilometer and even 100 m spatial resolutions and combined it with machine learning, optical snow cover information, and forest information to improve retrieval accuracy [12]. However, this method is still affected by surface scattering and soil freeze–thaw conditions. In areas with high soil moisture, backscatter variations may be affected by both snow accumulation and surface scattering changes, making it difficult to isolate the snow depth signal [13]. Therefore, SAR phase information provides a complementary approach for mountain snow depth retrieval [14].

Differential interferometric synthetic aperture radar (D-InSAR) measures interferometric phase differences between repeated SAR acquisitions. In dry snow, refraction within the snowpack produces an additional phase delay. This phase can be expressed in terms of snow water equivalent change [14,15] or converted to snow depth when density-dependent permittivity and local incidence angle are specified [16,17]. Thus, snow depth is a model retrieval from interferometric phase rather than a direct radar measurement. Previous studies have demonstrated the potential of this method for estimating snow depth [18]. However, its application in complex mountainous terrain remains challenging because retrieval accuracy depends on interferometric coherence, phase-unwrapping quality, and the density used in the phase-to-depth conversion [19]. Strong terrain relief and snowmelt processes may further reduce phase stability [20]. In addition, most previous snow depth studies used field or station measurements for validation. Few studies have directly compared snow depth retrieved from interferometric phase with UAV-LiDAR snow depth maps, especially in mountain areas with shallow snow, short snow duration, and complex terrain.

To address this gap, this study evaluates the applicability and uncertainty of Sentinel-1 D-InSAR for multi-temporal snow depth retrieval in complex mountainous terrain. We use the Maxian Mountains as a case study and retrieve snow depth from Sentinel-1 interferometric pairs acquired between 2020 and 2022. UAV-LiDAR snow depth estimates are used as independent reference data to assess the retrieval accuracy. More specifically, this study addresses three questions: (1) Can Sentinel-1 D-InSAR characterize the main spatial distribution of mountain snow depth? (2) How reliable are the retrieval results when evaluated against UAV-LiDAR snow depth estimates? (3) How does retrieval reliability vary with interferometric coherence, elevation, land cover, snow density, and local incidence angle? Addressing these questions can clarify how D-InSAR snow depth retrieval accuracy varies across space and snow conditions, providing a basis for interpreting the retrievals in mountain areas.

## 2. Study Area and Data

### 2.1. Study Area

**Figure 1:**
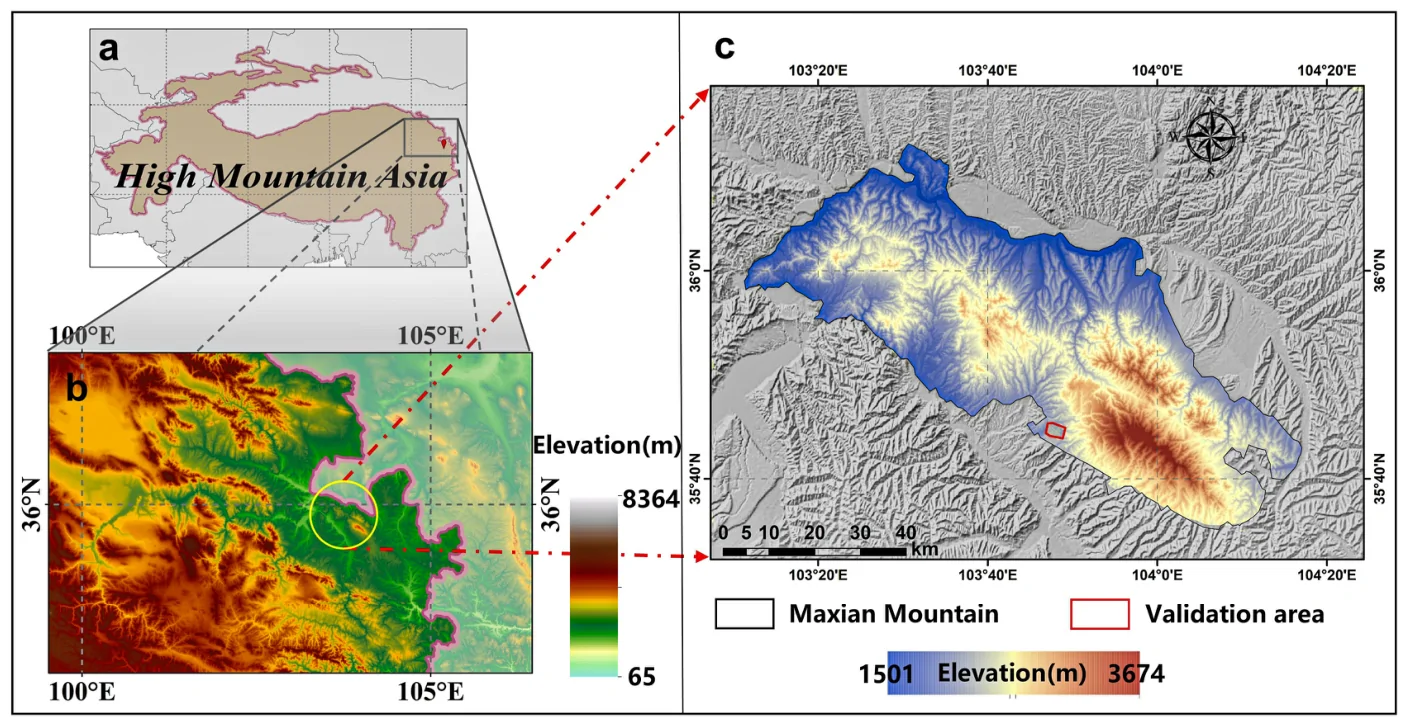
Location of Maxian Mountain within High Mountain Asia and the UAV-LiDAR validation area. (**a**) Location of the study region within High Mountain Asia. (**b**) Regional topographic setting of the Maxian Mountain area, and the color bar indicates elevation in meters. (**c**) Elevation distribution of Maxian Mountain, with the UAV-LiDAR validation area outlined in red.

Maxian Mountains is located on the eastern margin of High Mountain Asia [21], with elevations ranging from 1501 to 3674 m (Figure 1). Compared with the surrounding lowlands, Maxian Mountain has stronger topographic relief and more pronounced variations in local incidence angle and surface scattering conditions, making it suitable for evaluating Sentinel-1 D-InSAR snow depth retrieval in complex mountain terrain.

The study area has a subhumid climate and limited seasonal snow accumulation [22]. Snow cover is generally shallow and short-lived across the study area, but deeper snow tends to persist in the colder summit areas in the east [23]. Meteorological records near the summit from 2010 to 2020 show a maximum snow depth of approximately 40 cm, a mean annual air temperature of −2.0 to −0.7 °C, and a mean annual precipitation of 318.6 ± 54.3 mm. The strong elevation gradient, rugged terrain, vegetation differences, and freeze–thaw processes produce marked spatial heterogeneity in snow depth after snowfall events, increasing the uncertainty of D-InSAR retrieval and UAV-LiDAR-based validation.

### 2.2. Sentinel-1 SLC and UAV-LiDAR Reference Data

Sentinel-1 single-look complex (SLC) data were used for D-InSAR snow depth retrieval [8]. The SLC product preserves the amplitude and phase information of the radar signal and is suitable for interferometric processing, phase-change extraction, and coherence analysis. All Sentinel-1 SLC data were downloaded from the Alaska Satellite Facility Distributed Active Archive Center (ASF DAAC) Data Search/Vertex platform https://search.asf.alaska.edu/ (accessed on 14 July 2026) [24]. To reduce phase differences related to different viewing geometries, only acquisitions from the same relative orbit were used to form interferometric pairs.

All acquisitions were C-band Level-1 single-look complex products in Interferometric Wide swath mode, acquired using Terrain Observation with Progressive Scans SAR in dual polarization (VV/VH). The C-SAR center frequency is 5.405 GHz (λ = 5.547 cm). Interferometric Wide swath mode comprises three sub-swaths with transmitted range bandwidths of 56.5 MHz (IW1), 48.3 MHz (IW2), and 42.8 MHz (IW3), a 250 km swath, and a nominal single-look ground resolution of approximately 5 m × 20 m (range × azimuth).

Selection of the same relative orbit reduces geometric decorrelation but does not imply identical acquisition trajectories. During interferometric processing, the snow-covered SLC was co-registered to the snow-free reference geometry, and orbit information and the Copernicus DEM GLO-30 were used to model and remove phase contributions associated with the acquisition geometry and topography before phase unwrapping. The unwrapped phase and coherence layers used for snow-depth retrieval were represented on a common grid with a spacing of approximately 20 m. The snow retrieval was therefore performed using the residual unwrapped interferometric phase.

The interferometric pairs were selected using Landsat 8 optical imagery and field observation records. The reference acquisition represented the area snow-free before snowfall, whereas the snow acquisition was acquired after snowfall. Four interferometric pairs were processed, as listed in Table 1.

The UAV-LiDAR reference snow depth data were obtained from our previous study [21]. Repeat surveys covered the same area under snow-free and snow-covered conditions. The flight settings, including flight altitude, were kept as consistent as practicable among the surveys to maintain comparable sampling geometry and sufficient point density. Snow depth was calculated at corresponding 0.25 m grid cells by subtracting the georeferenced snow-free digital surface model from each snow-covered digital surface model. Thus, snow depth was determined from ground-referenced surface elevations rather than directly from platform altitude or raw sensor-to-surface range.

For temporal matching, the D-InSAR retrieval acquired on 24 November 2020 was paired with the contemporaneous UAV-LiDAR survey. For the April validation, the UAV-LiDAR survey was conducted on 8 April 2021, between the D-InSAR retrievals acquired on 5 and 11 April. Because no Sentinel-1 acquisition was available on the UAV-LiDAR survey date, the two bracketing retrievals were averaged to approximate the snow conditions on 8 April. The UAV-LiDAR maps were resampled to the common approximately 20 m D-InSAR grid before comparison. For comparison, both products were expressed as vertical snow depth. UAV-LiDAR derived this quantity from snow-covered and snow-free surface elevations, whereas the D-InSAR model converted the snow-induced slant-range phase delay to vertical snow depth through the refraction geometry in Equations (3)–(6).

### 2.3. Auxiliary Data

USGS Landsat 8 Collection 2 Level-2 surface reflectance products were used as optical auxiliary data [25]. These products are publicly available through USGS EarthExplorer https://earthexplorer.usgs.gov/ (accessed on 14 July 2026). Optical composites were generated using the Blue, SWIR1, and SWIR2 bands to visually identify snow-covered areas and qualitatively assess the spatial plausibility of the D-InSAR snow depth retrievals [26]. The composites were used only to provide qualitative snow-cover context for the D-InSAR maps and were not used to evaluate retrieval accuracy or validate the retrieved snow-depth values. The Landsat images were screened for cloud and cloud-shadow contamination before the optical composites were generated.

Topographic information was provided by the GLO-30 product, with a spatial resolution of approximately 30 m. The product is publicly available through the Copernicus Data Space Ecosystem (https://doi.org/10.5270/ESA-c5d3d65). The DEM was used to derive the local incidence angle and DEM hillshade.

Land-cover data were obtained from the Dynamic World product [27]. For each retrieval period, temporally matched Dynamic World observations were used to generate a land-cover classification map, which was then used to stratify interferometric coherence values and compare their distributions among different land-cover classes.

## 3. Methods

### 3.1. D-InSAR Snow Depth Retrieval

Sentinel-1 operates as a monostatic SAR system, whereas D-InSAR is a repeat-pass interferometric processing technique rather than a distinct radar acquisition mode. In this study, each interferogram was generated from a snow-free reference SLC and a co-registered snow-covered SLC acquired 6 or 12 days later along the same relative orbit. The resulting interferometric phase represents the phase difference between the two acquisitions rather than the absolute phase of either image. The physical basis of D-InSAR snow depth retrieval is the change in the two-way electrical path length caused by radar propagation through the snowpack [15]. During Sentinel-1 interferometric processing, a coherence threshold of 0.30 was applied as a quality-control criterion. Pixels with coherence below 0.30 were masked and treated as NoData before snow depth retrieval. The threshold was not derived as a universal physical boundary or demonstrated to be optimal for snow depth retrieval. A formal sensitivity analysis of alternative coherence thresholds was not performed.

**Figure 2:**
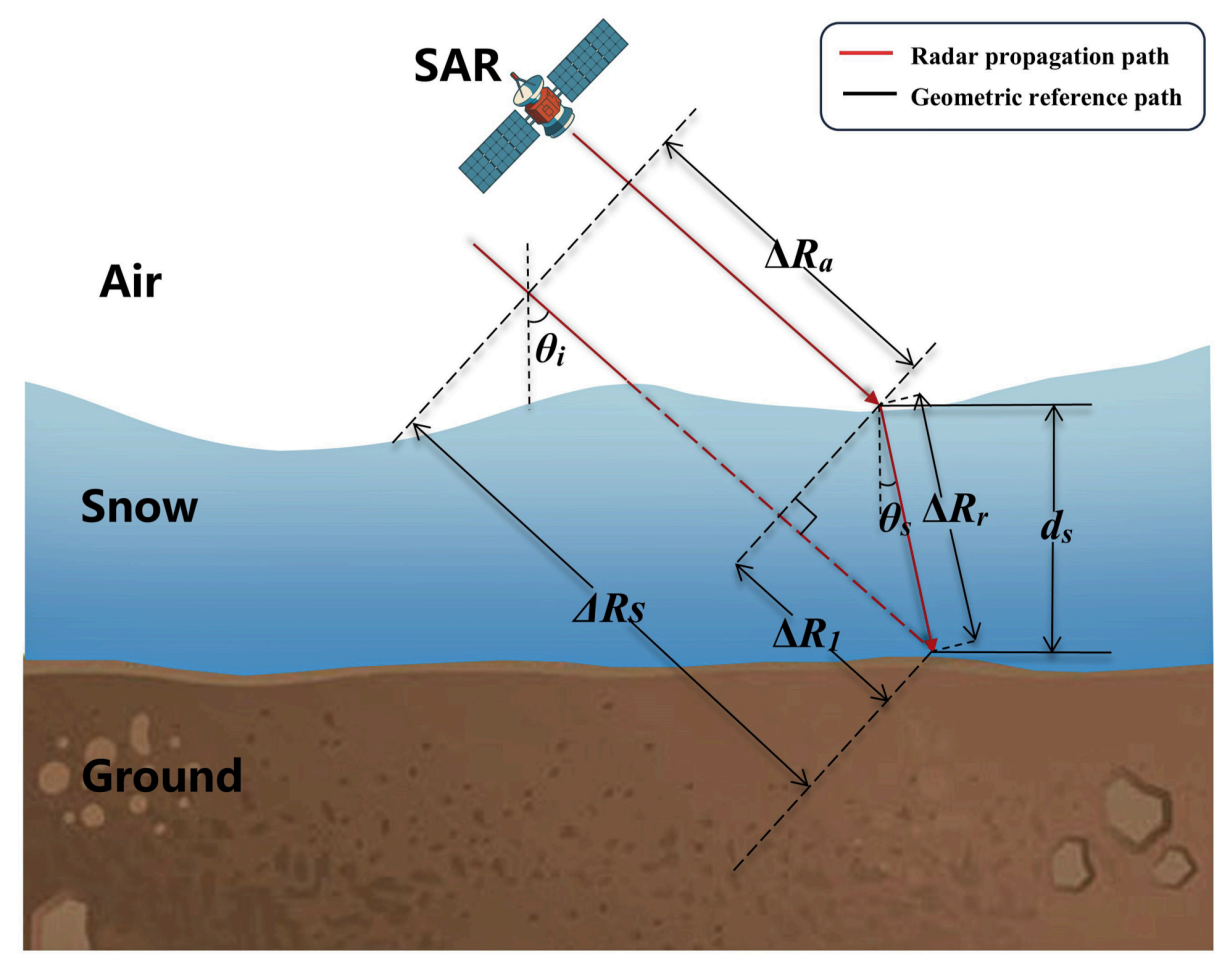
Geometric principle of snow depth retrieval using D-InSAR. The red line indicates the radar propagation path, and the black line indicates the geometric reference path.

As shown in Figure 2, under snow-free conditions, the radar signal propagates through the atmosphere to the ground surface and returns to the sensor. Under snow-covered conditions, the signal is refracted at the air–snow interface, propagates through the snowpack, and returns along the reciprocal path after scattering at the snow–ground interface. The resulting difference in electrical path length produces the snow-induced interferometric phase change.

This path difference produces an equivalent slant-range change and leads to an interferometric phase change [16,17]. The snow-induced phase change at pixel *i* can be expressed as

(1) $$\varphi_{i}^{\mathrm{snow}}=\psi_{i}^{1}-\psi_{i}^{2}=-\frac{4\pi }{\lambda }\Delta R_{i}$$

where λ is the Sentinel-1 C-band radar wavelength, set to 5.547 cm in this study; $\psi_{i}^{1}$ − $\psi_{i}^{2}$ is the unwrapped interferometric phase difference between the co-registered snow-free and snow-covered acquisitions, rather than the absolute phase of either acquisition; and ΔR_i_ is the snow-induced equivalent slant-range change at pixel *i*. The coefficient in Equation (1) accounts for the two-way radar propagation. The path lengths used below are defined for the corresponding one-way geometry.

Under snow-free conditions, the one-way path is the geometric radar path to the ground. Under snow-covered conditions, the corresponding path is the sum of the air segment and the phase-equivalent electrical path within the snowpack. Their difference, following the phase convention in Equation (1), can be expressed as

(2) $$\Delta R_{i}=\Delta R_{s}-(\Delta R_{a}+\sqrt{\varepsilon_{s}}\Delta R_{r})=\Delta R_{1}-\sqrt{\varepsilon_{s}}\Delta R_{r}$$

where Δ*R_s_* denotes the geometric path length under snow-free conditions, Δ*R_a_* is the portion of the propagation path in air under snow-covered conditions, and Δ*R_r_* is the geometric length of the refracted path within the snowpack. Here, *ε_s_* is the relative permittivity of snow, $\sqrt{\varepsilon_{s}}\Delta R_{r}$ represents the phase-equivalent electrical path length within the snow. Based on the geometry shown in Figure 2, Δ*R*_1_ can be expressed as

Δ*R*_1_ = Δ*R_r_*cos(*θ_i_* − *θ_s_*) (3)

where *θ_i_* is the local incidence angle and *θ_s_* is the refraction angle within the snow.

The geometric length of the refracted path within the snowpack is related to snow depth by

(4) $$\Delta R_{r}=\frac{d_{i}^{s}}{\cos\theta_{s}}$$

where $d_{i}^{s}$ is the snow depth at pixel *i*.

Refraction at the air–snow interface follows Snell’s law. Assuming that the refractive index of air is approximately unity, the refraction angle can be expressed as

(5) $$\sin\theta_{s}=\frac{\sin\theta_{i}}{\sqrt{\varepsilon_{s}}}$$

Finally, the snow depth at pixel *i* can then be expressed as

(6) $$d_{i}^{s}=-\frac{\lambda }{4\pi }\;\frac{\varphi_{i}^{\mathrm{snow}}}{\cos\theta_{i}-\sqrt{\varepsilon_{s}-\sin^{2}\theta_{i}}}$$

The relative permittivity of dry snow was estimated from snow density using the following empirical relationship [28]:

*ε_s_* = 1 + 1.6*ρ* + 1.86*ρ*^3^ (7)

where *ρ* is snow density. Equations (6) and (7) show that snow depth is calculated from interferometric phase using density-dependent permittivity and local incidence angle. When field measurements were unavailable, a dry-snow density of 0.18 g/cm^3^ was adopted from previous Sentinel-1 D-InSAR snow depth studies [17,29]. This is an empirical model input rather than a measured density for the corresponding acquisitions. Equation (7) describes dry snow and does not account for the dielectric effects of liquid water.

### 3.2. Accuracy Assessment and Uncertainty Analysis

To match the spatial scale of the Sentinel-1 retrievals, the high-resolution UAV-LiDAR snow depth maps were upscaled to the D-InSAR retrieval grid. The November 2020 dataset was used to assess retrieval accuracy under cold season conditions. The April 2021 dataset was used to examine the limitations of applying the dry-snow model under spring conditions. Agreement was assessed using *R*^2^, root mean square error (RMSE), and mean absolute error (MAE). Scatter plots and the linear fit were used to examine the relationship and any tendency toward overestimation or underestimation between the D-InSAR retrievals and the UAV-LiDAR reference data. These metrics describe grid-scale agreement within the sampled and coherence-screened area.

The uncertainty of the D-InSAR snow depth retrievals was analyzed mainly in terms of coherence, topographic conditions, land-cover type, and model parameters [20]. First, coherence maps were generated for each Sentinel-1 image pair, and the spatial differences in coherence among different periods were compared. Subsequently, DEM data and Dynamic World land-cover data were used to analyze coherence variations across elevation intervals and land-cover types. These statistics included all valid coherence pixels in the study area rather than only independently identified snow-covered pixels. The Dynamic World snow class was used only as a descriptive land-cover category and was not treated as an independent snow mask or as validation of the D-InSAR snow depth retrievals. In addition, the effects of snow density and local incidence angle were assessed by varying each parameter separately while keeping the other parameters constant.

## 4. Results

### 4.1. Spatial Distribution of Multi-Temporal D-InSAR Snow Depth Retrievals

**Figure 3:**
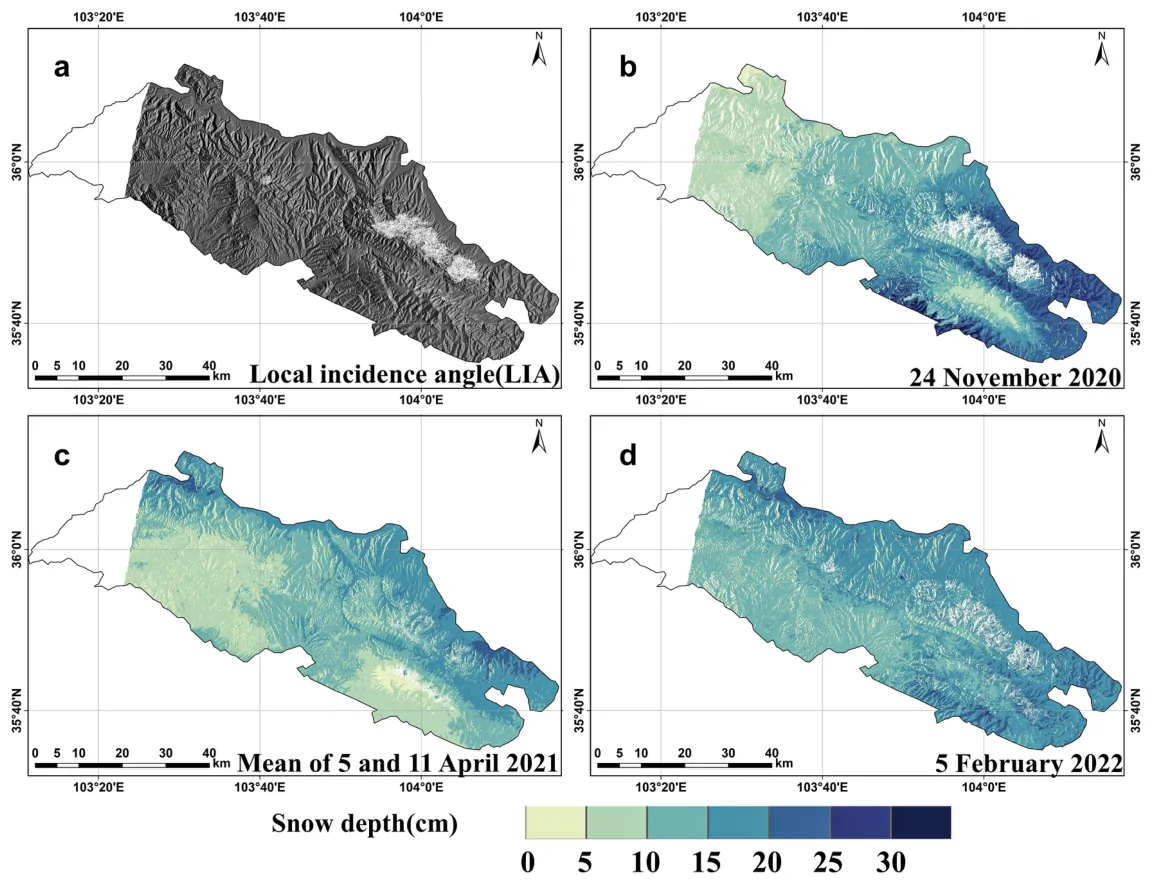
Local incidence angle and multi-temporal D-InSAR snow depth retrievals in Maxian Mountain. (**a**) Local incidence angle. (**b**–**d**) Snow depth retrieved from Sentinel-1 D-InSAR on 24 November 2020, the mean of 5 and 11 April 2021, and 5 February 2022. The Sentinel-1 Interferometric Wide swath single-look complex inputs have a nominal single-look ground resolution of approximately 5 m × 20 m (range × azimuth).

Figure 3 shows the local incidence angle and the D-InSAR snow depth retrievals for the three periods. The local incidence angle varied markedly across the study area. Furthermore, all three snow retrievals showed clear spatial heterogeneity, with higher snow depth mainly in the eastern mountains and lower values in the relatively flat western area. Specifically, high snow depth on 24 November 2020 was concentrated in the eastern mountains, with local values exceeding 25 cm. The April 2021 mean result showed lower snow depth overall and fewer high-value areas. On 5 February 2022, moderate snow depth was more continuously distributed.

**Figure 4:**
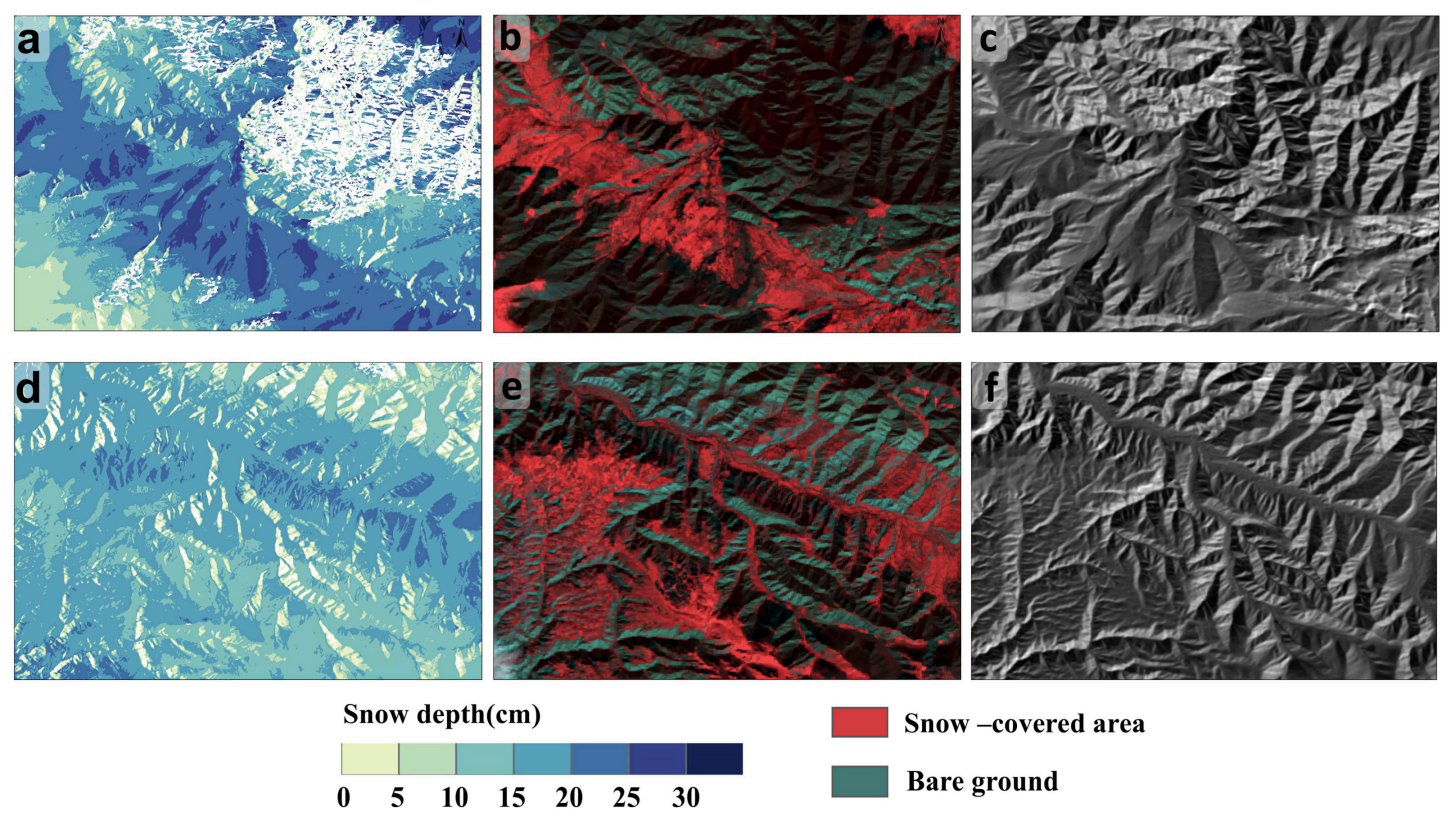
D-InSAR snow depth retrievals shown alongside Landsat 8 optical composites and DEM hillshade. (**a**,**d**) D-InSAR snow depth retrievals. (**b**,**e**) Landsat 8 surface reflectance composites using the Blue, Shortwave Infrared 1, and Shortwave Infrared 2 bands to distinguish snow-covered and snow-free areas. (**c**,**f**) DEM-derived hillshade. The optical composites provide snow-cover context only and do not validate the retrieved snow depth values.

Figure 4 presents the D-InSAR snow depth retrievals alongside the Landsat 8 optical composites and DEM hillshade. The optical composites provide qualitative information on the spatial extent of snow cover. Visual correspondence between snow cover and the retrieval maps does not establish the accuracy of the retrieved snow depth values. Local differences are visible along some snow-cover boundaries. These differences may be related to the spatial resolutions and sensing mechanisms of the two datasets, as well as the exclusion of pixels with coherence below 0.30. Some larger retrieved snow depth values were located in mountainous terrain, valleys, and gullies shown in the DEM hillshade. Local missing or noisy retrievals were also found, mainly in mountainous areas with strong terrain relief.

### 4.2. Accuracy Assessment Using UAV-LiDAR Reference Data

**Figure 5:**
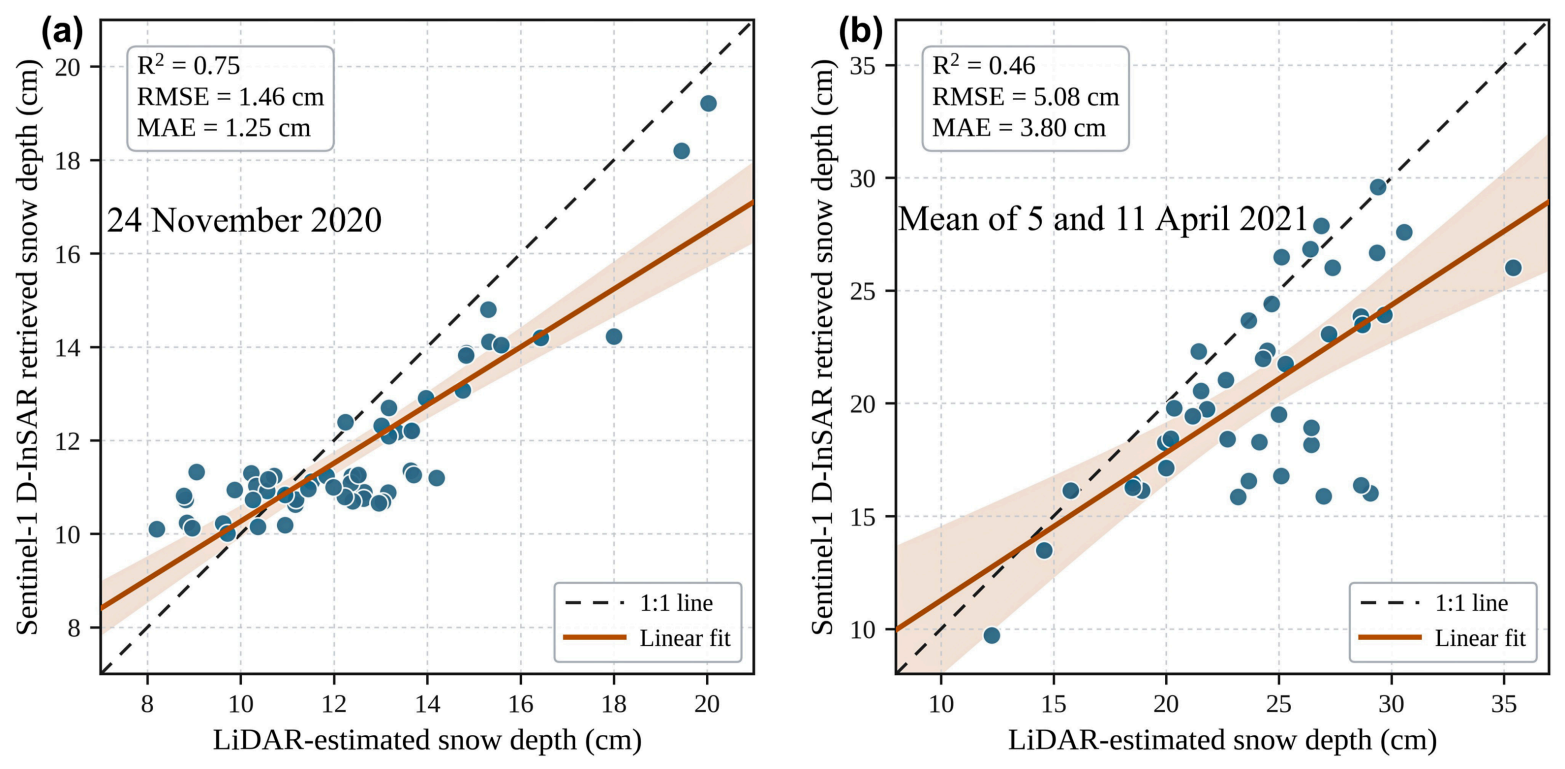
Validation of D-InSAR snow depth retrievals using UAV-LiDAR snow depth data. (**a**) Comparison for 24 November 2020. (**b**) Comparison for the mean of 5 and 11 April 2021. The dashed black line represents the 1:1 line, and the red line represents the linear fit. The shaded areas represent the 95% confidence intervals for the linear fits.

Figure 5 compares the D-InSAR snow depth retrievals with the UAV-LiDAR snow depth estimates. Overall, the two validation datasets showed a certain level of agreement, but the degree of agreement differed markedly. For the November 2020 shallow-snow dataset, the D-InSAR retrievals agreed well with the UAV-LiDAR reference data. The RMSE and MAE were 1.46 cm and 1.25 cm, respectively. In contrast, the April 2021 comparison showed greater scatter and disagreement, with RMSE and MAE values of 5.08 cm and 3.80 cm, respectively. In addition, most points in April lie below the 1:1 line, indicating a tendency toward underestimation relative to UAV-LiDAR. The November RMSE describes agreement for this particular grid-scale comparison and should not be interpreted as region-wide retrieval accuracy.

### 4.3. Interferometric Coherence and Retrieval Uncertainty

**Figure 6:**
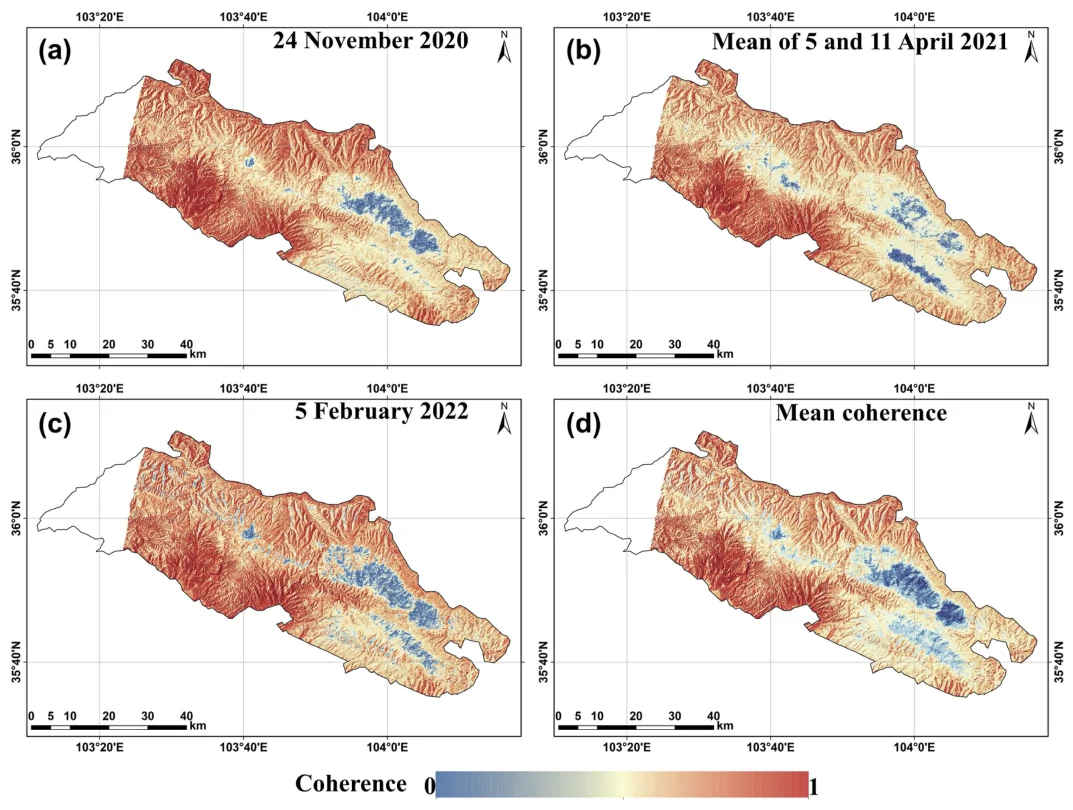
Spatial distribution of interferometric coherence for Sentinel-1 D-InSAR snow depth retrieval. (**a**) 24 November 2020. (**b**) Mean of 5 and 11 April 2021. (**c**) 5 February 2022. (**d**) Mean coherence across the three retrieval periods.

In this study, the three coherence maps showed similar spatial patterns (Figure 6). Coherence was generally higher in the western and lower-elevation parts of the study area and lower in the eastern mountains. The mean coherence map further showed that areas with low coherence were mainly located in mountainous terrain at high elevations with strong relief, where D-InSAR snow depth retrievals may have higher uncertainty. Coherence also varied among the retrieval periods. The 24 November 2020 and 5 February 2022 results showed relatively high coherence. In contrast, the April 2021 mean coherence was lower, with a larger extent of low coherence, especially in the eastern mountains at high elevations. This result is consistent with the lower validation accuracy of the April D-InSAR snow depth retrievals, suggesting that lower interferometric coherence may lead to greater retrieval uncertainty.

**Figure 7:**
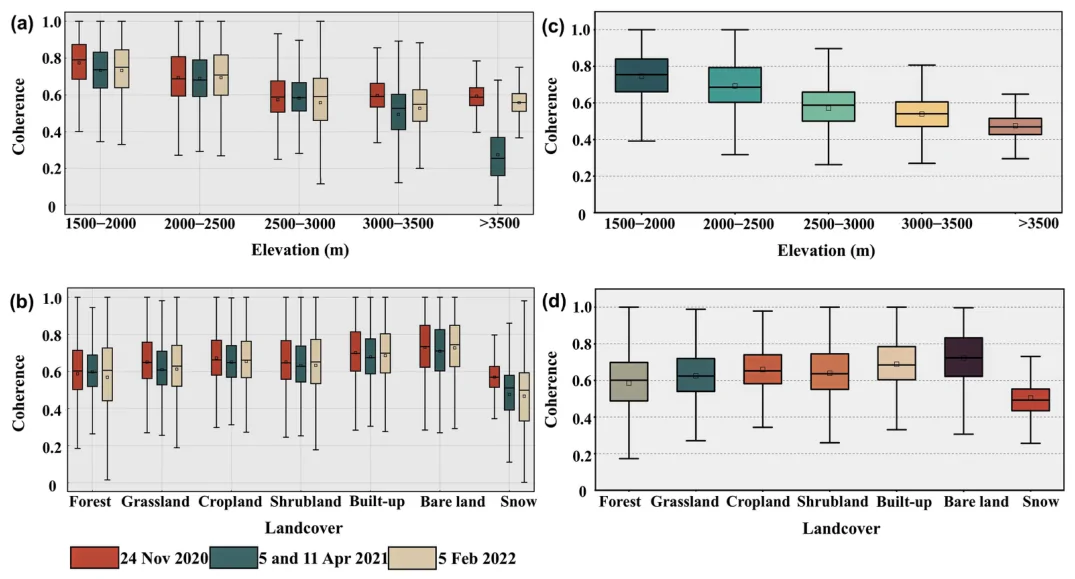
Variation in interferometric coherence with elevation and land cover. (**a**) Coherence distributions across elevation ranges for the three retrieval periods. (**b**) Coherence distributions across land cover types for the three retrieval periods. (**c**) Mean coherence across elevation ranges. (**d**) Mean coherence across land-cover types. The three retrieval periods are 24 November 2020, the mean of 5 and 11 April 2021, and 5 February 2022. The statistics include all valid coherence pixels in the study area rather than only snow-covered pixels.

Figure 7 shows how interferometric coherence varies across elevation intervals and land-cover types in the study area. Coherence decreased with elevation in all three periods, with the highest values at 1500–2000 m and the lowest values above 3500 m. The decrease was most pronounced in April 2021, especially above 3500 m. Furthermore, Coherence varied among land-cover types. Bare ground and built-up areas generally showed higher coherence, while the forest and snow land-cover classes showed lower coherence. These are land-cover-stratified coherence statistics, not land-cover-specific estimates of snow depth error. They do not isolate the effects of snow, vegetation, and terrain on retrieval errors. Because the analysis included all valid coherence pixels, these results describe regional coherence patterns rather than coherence specifically within independently identified snow-covered areas.

## 5. Discussion

### 5.1. Factors Influencing D-InSAR Snow Depth Retrieval Accuracy

The two comparisons with UAV-LiDAR data show different agreement across the two periods (Figure 5). The November RMSE was numerically lower than the 2.51 and 4.04 cm reported by Liu et al. [30] for Sentinel-1 D-InSAR retrievals before 3DVAR data fusion. The April RMSE was closer to the 5.74 cm reported by Singh et al. [31] using an improved Sentinel-1 D-InSAR model. These comparisons provide context, although observation conditions and validation designs differ. Within the present study, the larger April errors may partly reflect the rapid evolution of spring snow [18,20]. With increasing air temperature, snow in relatively flat areas may melt rapidly, while compaction and melt-refreeze processes can alter snow density, liquid water content, surface roughness, internal structure, radar penetration depth, and the effective phase center between SAR acquisitions. These changes can affect both the validity of the dry-snow phase-to-depth conversion in Equations (6) and (7) and the stability of the interferometric phase. However, snow temperature and liquid water content were not measured in this study. Therefore, the contributions of these processes cannot be quantified from the present dataset, and the April error metrics should be interpreted as retrieval performance under spring conditions rather than as validation of the dry-snow model.

For the April comparison, the mean of the D-InSAR retrievals from 5 and 11 April was used to represent the UAV-LiDAR survey date of 8 April. Same-day observations could not be obtained because of limited field accessibility and the fixed Sentinel-1 acquisition schedule. Because the survey date was midway between the two D-InSAR dates, the mean was used as a simple temporal interpolation between the two bracketing retrievals. However, this approach cannot fully account for rapid or nonlinear snowmelt [18]. Because daily snow depth observations were unavailable, the interpolation error could not be quantified separately. The April RMSE and MAE may therefore reflect both D-InSAR retrieval uncertainty and temporal mismatch, rather than errors caused by the D-InSAR method alone. Snow conditions and interferometric coherence also differed between the two periods. Their individual contributions to the larger April errors cannot be separated from the present comparison. The tendency toward underestimation in Figure 5b cannot be attributed to a single process. Liquid water, if present, may alter snow permittivity, radar penetration depth, the effective phase center, and interferometric coherence [20,32]. However, these effects do not establish the direction of the snow-depth bias, and their contributions cannot be separated without measurements of snow temperature and liquid water content.

Figure 7 shows that coherence decreased with elevation and was lower over forest than over bare ground. These statistics describe associations between coherence, elevation, and land cover. They do not establish a causal relationship between forest cover and snow depth errors. Mountainous terrain is more susceptible to geometric decorrelation. It is also affected by terrain-related distortions, including layover, shadowing, and foreshortening [33]. These effects can degrade interferometric phase quality. Snowpack changes and vegetation scattering can alter the interferometric phase and weaken the coherent contribution from the snow–ground interface [18,29,34]. In dense forest, the assumption of a stable snow–ground scattering interface may not hold. Passing the coherence threshold does not establish that a forest retrieval represents ground-referenced snow depth. Forest-specific error contributions were not quantified in this study. In spring, higher snow liquid water content may also increase the absorption and attenuation of C-band radar signals, making the interferometric phase less stable for snow depth retrieval [32].

### 5.2. Parameter Sensitivity of the D-InSAR Retrieval Model

**Figure 8:**
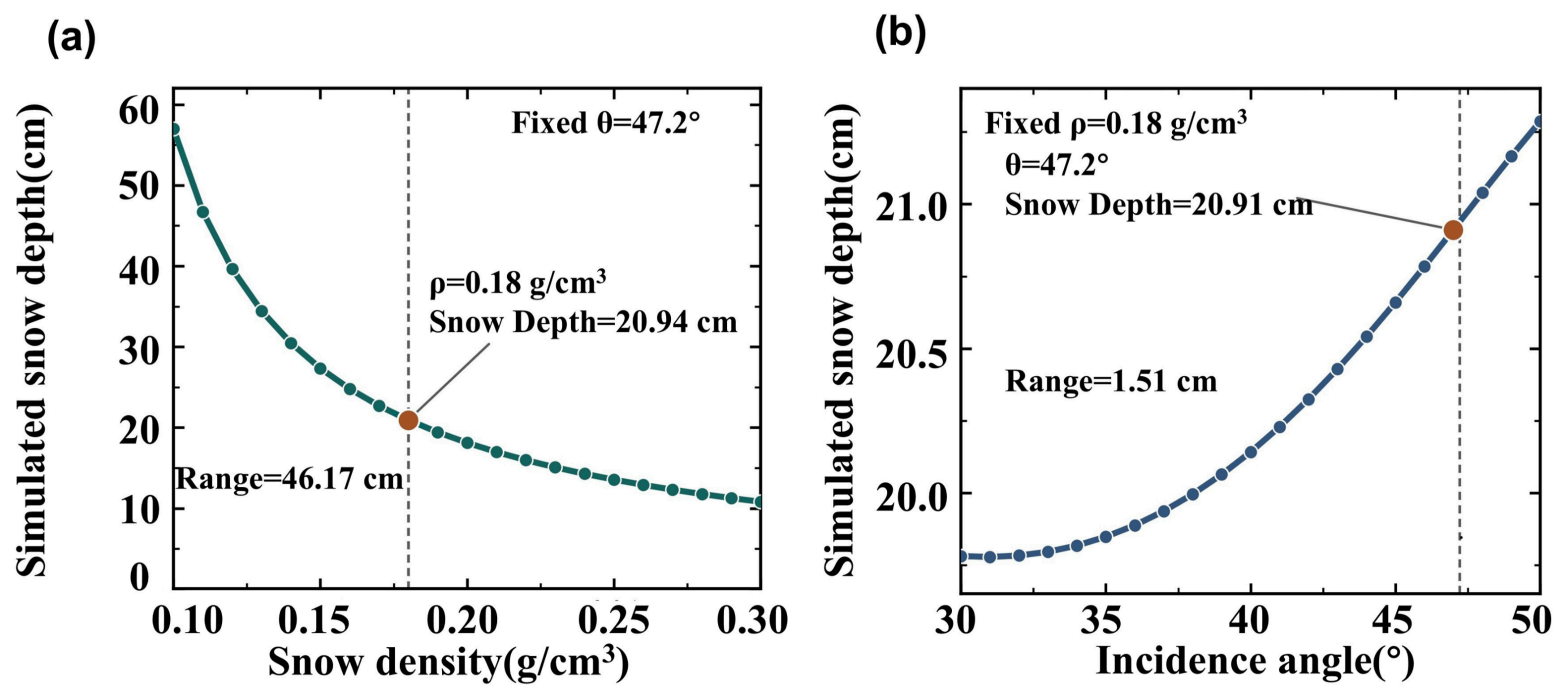
Parameter sensitivity of D-InSAR snow depth retrieval to snow density and local incidence angle. (**a**) Simulated snow depth as a function of snow density under a fixed local incidence angle of 47.2°. (**b**) Simulated snow depth as a function of local incidence angle under a fixed snow density of 0.18 g/cm^3^. The vertical dashed lines mark the reference parameter values, and the orange markers show the corresponding simulated snow depths.

Figure 8 shows that the D-InSAR snow depth retrieval model is sensitive to both snow density and local incidence angle, with snow density having the stronger effect. At fixed phase and local incidence angle, increasing density from 0.10 to 0.30 g/cm^3^ reduced the retrieved snow depth from 56.9 to 10.8 cm. The adopted value of 0.18 g/cm^3^ has been used as an empirical dry-snow density in previous Sentinel-1 D-InSAR snow depth studies, but it remains an empirical model input rather than a contemporaneous measurement of snow density in the study area. However, a uniform value does not remove density-related uncertainty or represent the full spatial and temporal variability caused by changes in air temperature, liquid water content, compaction, and melt-refreeze processes [35]. Consequently, the reported snow depth depends on the assigned density, and a substantial difference between the actual density and 0.18 g/cm^3^ may introduce a systematic scaling bias. Therefore, Figure 8a represents a conditional sensitivity analysis and should not be interpreted as the observed uncertainty range of the April retrievals. Because contemporaneous snow-density measurements were unavailable, the actual density-related bias cannot be quantified from the present dataset.

The local incidence angle also contributes to spatial variation in the retrieved snow depth. In the spatial retrieval, the local incidence angle was calculated for each pixel from the DEM and Sentinel-1 imaging geometry and used in Equation (6). Therefore, the variation caused by terrain shown in Figure 3a was retained in the snow depth retrieval. Figure 8b shows this effect by holding interferometric phase and snow density constant. Increasing the local incidence angle from 30° to 50° increased the simulated snow depth from 19.78 to 21.29 cm, a difference of 1.51 cm. The magnitude of this effect varies with interferometric phase, but its direct influence was smaller than that of snow density within the ranges examined. In areas with strong relief, errors in local incidence angle may interact with geometric distortions such as layover, shadowing, and foreshortening, thereby reducing retrieval accuracy [33].

Figure 8 presents a conditional sensitivity analysis rather than a complete estimate of retrieval uncertainty. Snow density and local incidence angle enter the conversion model directly, whereas coherence, terrain, and snow wetness affect phase quality, imaging geometry, or the validity of the dry-snow assumption. The endpoint values in Figure 8 should therefore not be interpreted as the observed uncertainty range of the April retrievals. Quantifying the actual density-related bias would require contemporaneous snow-density measurements.

### 5.3. Applicability and Limitations of D-InSAR Snow Depth Retrieval

This study shows that the D-InSAR method can characterize the main spatial distribution of mountain snow depth. The November 2020 dry-snow dataset showed better validation performance than the April 2021 dataset. Unlike empirical backscatter and machine learning models, D-InSAR relates interferometric phase to snow depth through a propagation model and does not require regional training data, although the conversion still depends on an assumed snow density. Therefore, it has potential to provide spatial snow-depth information under suitable dry-snow conditions. This information could complement optical snow-cover mapping and coarse-resolution passive microwave products [6,7]. The workflow can also be applied to other mountain regions under similar conditions, particularly for relatively thin, dry snow, short temporal baselines, stable snow–ground scattering, and sufficient interferometric coherence. Its performance may vary with regional snow, vegetation, and terrain conditions [17,29].

The quarter-wavelength criterion is not a fixed lower detection limit for snow depth in the propagation model used here. At *ρ* = 0.18 g/cm^3^ and *θ_i_* = 47.2°, Equation (6) indicates that a snow depth of 1.4 cm corresponds to a modeled phase change of approximately 0.61 rad (35°). This calculation describes the theoretical phase response. Reliable detection requires evaluating this response relative to phase noise and residual atmospheric, orbit, and DEM contributions. In addition, resampling the 0.25 m UAV-LiDAR data to the D-InSAR grid does not eliminate differences in spatial support. Therefore, the comparison represents grid-scale agreement between the D-InSAR retrievals and the aggregated UAV-LiDAR reference values, rather than individual 0.25 m snow-depth values. Subpixel variability may nevertheless affect the comparability of the two datasets. Although the two datasets were represented on the same grid, residual geolocation and co-registration errors were not independently quantified and may contribute. Their Agreement was assessed using R^2^, root mean square error (RMSE), and mean absolute error (MAE) disagreement, particularly in areas of steep terrain [36].

However, the reliability of D-InSAR depends strongly on local coherence and snow conditions [37]. Areas with low coherence are more prone to phase-unwrapping errors, missing retrievals, and local anomalies. The present data show that coherence varies with elevation and land cover. However, this study did not separately quantify the contributions of wet snow, vegetation scattering, and geometric distortion to snow depth retrieval errors. At the same time, uncertainties in snow density, local incidence angle, and DEM accuracy may affect the retrieved snow depth. Because pixels with coherence below 0.30 were excluded before snow depth retrieval, the comparison with UAV-LiDAR data was restricted to the retained pixels.

Although the modeled geometric and topographic phase components were removed, residual orbit errors may introduce phase ramps over broad spatial scales, while DEM errors may produce artifacts associated with terrain. A first-order estimate based on the perpendicular baselines of the interferometric pairs suggests that a DEM elevation error of 4–8 m would correspond to less than approximately 0.5 cm of equivalent snow depth error. This estimate does not include possible effects on local incidence angle or geolocation. Temporal decorrelation caused by changes in the snowpack, vegetation, or ground surface between acquisitions can reduce coherence and increase uncertainty in phase unwrapping. Spatially variable atmospheric delays cannot be separated reliably from snow-related and other residual phase contributions using a single interferogram. Errors in phase unwrapping may also produce local discontinuities and anomalous snow depth retrievals. The individual contributions of these errors were not quantified separately in this study and are therefore considered additional sources of retrieval uncertainty.

## 6. Conclusions

This study retrieved multi-temporal snow depth in the Maxian Mountains by converting the Sentinel-1 D-InSAR phase through a dry-snow propagation model using snow density and local incidence angle and independently evaluated the retrievals with UAV-LiDAR snow depth estimates. At the same time, we analyzed the sources of uncertainty in the D-InSAR method by considering interferometric coherence, elevation, land-cover type, snow density, and local incidence angle.

The results show that D-InSAR can characterize the main spatial distribution of snow depth in the study area, while retrieval performance varied with snow conditions and interferometric coherence. Compared with November 2020, the April 2021 comparison had an RMSE that was 3.62 cm larger, indicating greater disagreement under spring conditions. Possible wet-snow effects, vegetation cover, and complex terrain may reduce interferometric phase stability and increase retrieval uncertainty. The parameter sensitivity analysis showed that snow density has a substantial effect on the retrieval results, while the direct effect of local incidence angle was relatively small within the ranges examined. Therefore, the retrieved snow depth depends on the assigned density and should be interpreted as a model retrieval rather than a direct radar measurement. These findings provide practical guidance for selecting suitable observation conditions and interpreting D-InSAR snow depth retrievals in mountain environments. Accordingly, the April comparison represents retrieval performance under spring conditions rather than validation of the dry-snow formulation.

Future studies should test this workflow in other mountain regions using reference data acquired on the same day and covering larger areas. Concurrent measurements of snow density and liquid water content, together with improved quality control for phase unwrapping, would support multivariable uncertainty analysis and a more complete assessment of D-InSAR snow depth retrieval under different mountain conditions.

## Figures and Tables

**Table 1:** Sentinel-1 interferometric pairs used for D-InSAR snow depth retrieval.

Reference Acquisition (Pre-Snowfall)	Snow-Covered Acquisition (Post-Snowfall)	Perpendicular Baseline (m)	Temporal Baseline (Days)	Mean Coherence
18 November 2020	24 November 2020	−64.586	6	0.6789
24 March 2021	5 April 2021	−28.007	12	0.6625
30 March 2021	11 April 2021	58.525	12	0.6521
24 January 2022	5 February 2022	−9.464	12	0.6619

Note: Interferometric pairs were selected from available Sentinel-1 acquisitions using Landsat 8 imagery and field observations to identify snow-free reference and snow-covered secondary acquisitions, with temporal baselines limited to 12 days. The pairs represented different snow conditions, and the 2020 and 2021 snow-covered acquisitions were selected as close as possible to the UAV-LiDAR surveys.

## Data Availability

The original contributions presented in this study are included in the article. Further inquiries can be directed to the corresponding author.

## Abbreviations

The following abbreviations are used in this manuscript:

D-InSAR	Differential Interferometric Synthetic Aperture Radar
LiDAR	Light Detection and Ranging
MAE	Mean Absolute Error
RMSE	Root Mean Square Error
SAR	Synthetic Aperture Radar
SLC	Single-Look Complex
UAV	Unmanned Aerial Vehicle
